# A novel gelatin/carboxymethyl chitosan/nano-hydroxyapatite/β-tricalcium phosphate biomimetic nanocomposite scaffold for bone tissue engineering applications

**DOI:** 10.3389/fchem.2022.958420

**Published:** 2022-09-08

**Authors:** Qiushuo Sun, Lu Yu, Zhuocheng Zhang, Cheng Qian, Hongzhe Fang, Jintao Wang, Peipei Wu, Xiaojing Zhu, Jian Zhang, Liangjun Zhong, Rui He

**Affiliations:** ^1^ School of Stomatology, Hangzhou Normal University, Hangzhou, China; ^2^ Center of Stomatology, The Affiliated Hospital of Hangzhou Normal University, Hangzhou, China; ^3^ Institute of Life Sciences, College of Life and Environmental Sciences, Key Laboratory of Mammalian Organogenesis and Regeneration, Hangzhou Normal University, Hangzhou, China; ^4^ College of Materials, Chemistry and Chemical Engineering, Hangzhou Normal University, Hangzhou, China

**Keywords:** bone tissue engineering, nanocomposites, freeze drying, stirring foaming, morphological analysis

## Abstract

Hydroxyapatite (HA) and tricalcium phosphate (TCP) constitute 60% of the content of the bone, and their combination has a better effect on bone tissue engineering than either single element. This study demonstrates a new degradable gelatin/carboxymethyl chitosan (CMC) bone scaffold loaded with both nano-HA and β-TCP (hereinafter referred to as HCP), and freeze drying combined with stir foaming was used to obtain highly connected macropores. Only a few studies have used these components to synthesize a four-component osteogenic scaffold. The aim of this study was to comprehensively assess the biocompatibility and osteoinductivity of the nanocomposites. Three HCP/CMC/gelatin scaffolds were made with different HCP contents: group A (10 wt% HCP), group B (30 wt% HCP), and group C (50 wt% HCP) (the ratio of nano-HA and β-TCP was fixed at 3:2). The scaffolds were macroporous with a high porosity and pore connectivity, as observed by morphological analysis by scanning electron microscopy. Additionally, the pore size of groups A and B was more homogeneous than that of group C. There were no significant differences in physicochemical characterization among the three groups. The Fourier-transform infrared (FTIR) spectroscopy test indicated that the scaffold contained active groups, such as hydroxyl, amino, or peptide bonds, corresponding to gelatin and CMC. The XRD results showed that the phase structures of HA and β-TCP did not change in the nanocomposite. The scaffolds had biodegradation potential and an appreciable swelling ratio, as demonstrated with the *in vitro* test. The scaffolds were cultured *in vitro* with MC3T3-E1 cells, showing that osteoinduction and osteoconduction increased with the HCP content. None of the scaffolds showed cytotoxicity. However, cell adhesion and growth in group B were better than those in group A and group C. Therefore, freeze drying combined with a stir foaming method may have a solid component limit. This study demonstrates a novel four-component scaffold *via* a simple manufacturing process. Group B (30% HCP) had the best characteristics for bone scaffold materials.

## 1 Introduction

Bone tissue engineering has been studied to repair critical bone defects for many years because there are many limitations to autologous bone graft (Roseti et al., 2017; Shao et al., 2017). The optimal scaffold for bone regeneration should have porosity, highly connected macropores, and an appropriate pore size to ensure osteoconduction, vascularization, and osteointegration (Wu et al., 2017; Yan et al., 2019), respectively. Additionally, the degradation of the scaffold should match the osteogenesis process with minimal changes to the surrounding tissue when metabolized (Bharadwaz and Jayasuriya, 2020).

Inorganic minerals comprise 65% of the content of bone, and the main component is calcium phosphate (CaP) (Buck and Dumanian, 2012). Hydroxyapatite (HA), which has a similar chemical composition and crystal structure as the main inorganic mineral in the human bone tissue, is biocompatible. Compared with other biomaterials, HA has many advantages, including biological activity and selectivity to cancerous cells, which makes its application to tissue engineering promising (Ghiasi et al., 2020; Lowe et al., 2020). HA composition in humans is similar to that of nano-HA, with better biological properties than its bulk counterpart (Venkatesan and Kim, 2014; Lowe et al., 2020). The surface area of nanoparticles per unit mass is significantly larger than micron particles, which increases the number of atoms on the surface and improves the particle activity. These characteristics are beneficial for healing tissues (Vieira et al., 2017; Abdul Halim et al., 2021). However, the low degradability of HA may lead to bone deformities and increase the risk of fracture around HA bone implants (Bohner et al., 2020). Additionally, the scaffold must allow for the migration and growth of cells to maintain a stable structure (Noor, 2013). To address this issue, many researchers use natural degradable materials, such as gelatin or hyaluronic acid (Catalan et al., 2019; Nabavinia et al., 2019; Samirah et al., 2021; Xu et al., 2021). It is common to use organic compounds with nano-HA to form three-component scaffolds such as gelatin/chitosan/nano-HA scaffolds (Filippi et al., 2020). Although organic polymer compounds provide some degradability to the scaffolds, the residual nano-HA still hinders bone formation (Oryan et al., 2017). Alternatively, the combination of β-tricalcium phosphate (TCP) and HA has better biological properties for bone formation than each of the components alone (Oryan et al., 2017; Da Silva Brum et al., 2019; Da Silva Brum et al., 2021). However, there are few studies using both natural polymer bone scaffold materials. The advantages of β-TCP are its biodegradability approaching that of bone mineral, it can be resorbed by osteoclasts and macrophages, and it provides Ca and P in quantities such that Ca exceeds the threshold necessary to form the bone (Yamada et al., 1997; Jensen et al., 2009). However, the rapid degradation of β-TCP and its low mechanical strength do not provide a stable initial environment in the bone defect area, which reflects the advantages of HA (Bohner et al., 2020). These two CaPs have been used as biphasic CaP for many years (Cho et al., 2010). However, their ratio is adjusted by controlling the sintering temperature and pH of the reaction system, which is more complex than using natural polymers to directly envelope and crosslink the CaP particles (Ebrahimi et al., 2017). Natural polymers are more convenient for studying the influence of the ratio on the scaffold performance.

This study aimed to fabricate a scaffold for bone recovery of a nonweight bearing area. It is not common to apply two CaPs (nano-HA and β-TCP) to a natural polymer scaffold at the same time. Using freeze drying and a stir foaming method, the selected nano-HA, β-TCP, gelatin, and carboxymethyl chitosan (CMC) were evaluated to improve the classic three-component scaffold that typically contains nano-HA or β-TCP and produce a four-component bone scaffold material with better biological performance. The phase structure, surface structure, swelling proportion, biodegradation, and mechanical characteristics of the nanocomposite were demonstrated. *In vitro* cell feasibility tests were used to evaluate the scaffold biocompatibility for osteoblast (MC3T3-E1) adhesion, proliferation, and osteogenic differentiation.

## 2 Materials and methods

### 2.1 Fabrication of scaffolds

Three groups of nano-HA and β-TCP (hereinafter referred to as HCP)/CMC/gelatin scaffolds were made with different HCP contents. Group A (10 wt% HCP), group B (30 wt% HCP), and group C (50 wt% HCP) had 0.17, 1.08, and 2.50 g more HCP, respectively [the ratios of nano-HA (Macklin, Beijing, China) and β-TCP (Macklin) were fixed with 3:2 HCP], in 38 ml of double distilled water. The solution was mixed by ultrasonic shaking for 1 h. An amount of 2 g of gelatin (Macklin) and 0.5 g of CMC (Solarbio, Beijing, China) were dissolved into the mixture at 50°C, and the mixture was mixed at 300 rpm for 12 h to ensure complete dissolution. Freeze drying combined with high-speed stirring was used according to the procedure described by Maji et al. (2018). The solution was stirred using a high-speed blender at 5,000 rpm for 5 min until the foam height was unchanged. The foamed mixture was transferred to 24-well culture plates, frozen at −80°C for 12 h, and then freeze dried for 48 h. The scaffold was soaked in a 0.2 wt% glutaraldehyde solution for 1 h to crosslink. The scaffold was washed with a deionized water solution with sodium borohydride to remove residual glutaraldehyde, washed with deionized water again, and air-dried.

### 2.2 Physicochemical characterization of the macroporous scaffold

#### 2.2.1 Morphological analysis

A scanning electron microscope (SEM) (S-4800, HITACHI, Chiyoda, Japan) was used to observe the microstructures in the nanocomposites. Samples from each group were sputter-coated with gold and visualized at 30 kV. The SEM images were processed by Photoshop 6.0 and then imported into ImageJ for further processing. Briefly, the scale was set (approximately 301 pixels in an image was 1 mm of the actual sample), the threshold was adjusted to match the red areas covering the pores in the images, and the particles were analyzed to determine the pore size of the scaffold finally.

#### 2.2.2 Evaluation of surface chemical properties

The scaffolds were air-dried and ground into a powder with a tungsten steel drill bit to make test samples. X-ray diffraction spectroscopy (XRD) (D8, Bruker, Billerica, Massachusetts, United States) was used to study the crystal structures of these nanocomposites with a CuKα radiation source operating at a tube power of 3 kW. Data were collected in the scanning range from 2θ = 0° to 120° at a step size of 0.0001°/min. Fourier-transform infrared (FTIR) (Nicolet iS5, Thermo Fisher Scientific, Waltham, MA, United States) spectra were recorded over a wavenumber range of 4,000–400 cm^−1^ (resolution of 4 cm^−1^) using the KBr method.

### 2.3 Porosity measurement

The porosity of the scaffold was measured using the liquid displacement method described by Han et al. (2014). The scaffold was immersed in a known volume (*Va*) of absolute ethanol for 1 h to ensure that the inside of the scaffold was filled with liquid, and the total volume was recorded (*Vb*). The scaffold impregnated with ethanol was removed, and the residual volume of ethanol (*Vc*) was recorded. The porosity of the scaffold was calculated using the following formula:

$$Porosity\left(\%\right)=\frac{\left(Va-Vc\right)*100}{\left(Vb-Vc\right)}.$$

### 2.4 Mechanical test

The scaffolds were cut into a cylindrical shape with an approximate 10 mm diameter and 10 mm height using a high-speed dental handpiece. A universal testing machine (Instron5966, Instron, Boston, MA, United States) was used to test the mechanical properties of the nanocomposite with a saturation compression speed of 5 mm/min. Young’s modulus was calculated using the stress–strain curve (Maji et al., 2018).

### 2.5 Swelling ratio of the scaffolds

A swelling ratio test was used with the methods described by Montaser et al. (2021). A 100 mg sample of each group was used to test the scaffold dry weights (*W2*). Each sample was immersed in a tube containing 50 ml of PBS (pH 7.4) and incubated at 37°C. The wet weight (*W1*) of the scaffold was measured after the removal of excess surface water by gently blotting with an aseptic towel every 6 h until the weight did not change. The swelling ratio of the scaffold was calculated using the following equation:

$$\text{Swelling ratio}\left(\%\right)=\frac{\left(W1-W2\right)*100}{W1}\;.$$

### 2.6 Biodegradation potential of the scaffolds

The protocol described by Agarwal et al. (2016) was used. Initially, 100 mg of the scaffold from each group was measured for the initial wet weight (*Wa*), as described in Section 2.5. These samples were soaked in PBS (pH 7.4) containing 0.01% (w/v) collagenase type I (BioSharp, Beijing, China). The wet weight (*Wb*) after degradation was measured at 1, 3, and 7 days. The percentage degradation (D_x_) of the scaffold was calculated using the following equation:

$$Dx\left(\%\right)=\frac{\left(Wa-Wb\right)*100}{Wa},\;X=A,B,C.$$

### 2.7 Cell culture studies

#### 2.7.1 Cell culture

A mouse calvarial preosteoblast cell line (MC3T3-E1 Subclone 4, ATCC-LGC, standards, MeisenCTCC, Zhejiang, China) was cultured in α-MEM medium (CellMax, Beijing, China) supplemented with 10% fetal bovine serum (CellMax) and 1% penicillin–streptomycin solution (Solarbio) in a humidified 37°C incubator with 5% CO_2_. The culture medium was changed every 2–3 days. Cells between passages 5 and 9 were used for all experiments.

#### 2.7.2 Cytotoxicity assay

The cytotoxicity of the nanocomposite scaffolds was assessed using a MTT (3-(4,5dimethylthiazol-2-yl)-2,5-diphenyltetrazolium bromide) assay (Beyotime, Shanghai, China). To assay the mitochondrial reduction of MTT, the scaffolds of groups A, B, and C were ground into powder on a clean bench and irradiated with an ultraviolet lamp for 5 h. The scaffold (0.1 g/ml) was added to the complete medium and incubated at 37°C for 72 h. Leach liquors released from the samples were used for the cytotoxicity assay (Chen et al., 2019). The cells were cultured for 24, 48, and 72 h to evaluate the viability, and a complete α-MEM medium was used for the control. The OD value of each well was read at a wavelength of 490 nm using a microplate reader (SpectraMax iD5, Thermo Fisher Scientific). Every assay was repeated three times. The cell viability was calculated using the following equation:

$$\text{Cell viability}\left(\text{\%}\right)=\frac{\text{OD}\left(\text{Experimental}\right)-\text{OD}\left(\text{Blank}\right)}{\text{OD}\left(\text{Control}\right)-\text{OD}\left(\text{Blank}\right)}\text{∗}100\text{,}$$

where OD (experimental) was the value from the experimental group, OD (blank) was the value from the blank well, and OD (control) was the value from the medium control. Cytotoxicity was assessed according to ISO 10993-5-09.

#### 2.7.3 Cell adhesion and proliferation assays

The scaffolds from each group were cut to approximately 5 mm in diameter and 1 mm in height using a high-speed dental handpiece. The samples were sterilized by ultraviolet radiation and washed three times with DPBS before the cell seeded. A Fuchs-Rosenthal counting chamber was used to adjust the cell density to 5×10^4^ cells/ml. To prevent the cells from being washed into the well plate before adhering to the scaffold, 100 μl of the cell suspension was placed on both sides of the scaffold with 200 μl α-MEM medium, and then, 300 μl α-MEM medium was added to each well after 4 h. These cell-scaffold composites were incubated in a 24-well sterile tissue culture plate, and the medium was replaced every day. Cell adhesion on the 2nd day and cell proliferation on the 3rd and 5th day were tested (Kazimierczak et al., 2019). These cell-scaffold composites were washed with PBS to remove the unattached or dead cells, and then, the composites were fixed with 4% paraformaldehyde. The samples were stained with TRITC phalloidin-594 (Yeasen, Shanghai, China) and DAPI (Solarbio) for imaging with an inverted fluorescence microscope. Five visual fields were randomly selected to count cell nuclei by ImageJ software and estimate cell proliferation (Kazimierczak et al., 2019).

#### 2.7.4 Alkaline phosphatase activity

MC3T3-E1 cells were cultured in an osteogenic medium to study *in vitro* culture differentiation toward the osteogenic lineage and characterization. Cell-scaffold composites of each group described in the previous section and a scaffold containing only gelatin and CMC as a control group were fabricated. After differentiation was induced for 7 and 14 days, the ALP activity was measured using an ALP assay kit (Beyotime, Shanghai, China).

### 2.8 Statistical analysis

The results were reported as the mean ± standard deviation (SD). A one-way analysis of variance (ANOVA) was analyzed to evaluate the statistics of the data in SPSS 26.0.

## 3 Result

### 3.1 Morphological analysis

**FIGURE 1:**
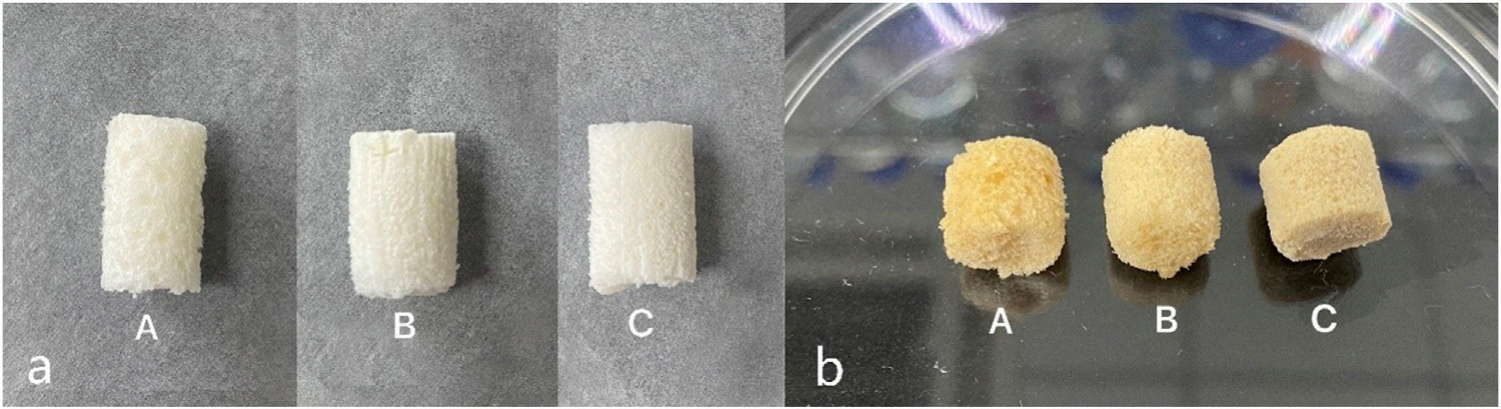
Macro appearance of the scaffold **(a)** before and **(b)** after crosslinking with glutaraldehyde. Images from the left to right are group A (10% HCP), group B (30% HCP), and group C (50% HCP). The scaffolds were white to pale yellow and translucent with a porous morphology even after crosslinking with glutaraldehyde.

The nanocomposite macroporous scaffolds were synthesized using a stir foaming and freeze drying method. In this process, the amine groups from both the gelatin and CMS crosslink with glutaraldehyde in the scaffolds, the scaffolds appearance changed from white to pale yellow, and the scaffolds were translucent with porous morphology (Figure 1).

**FIGURE 2:**
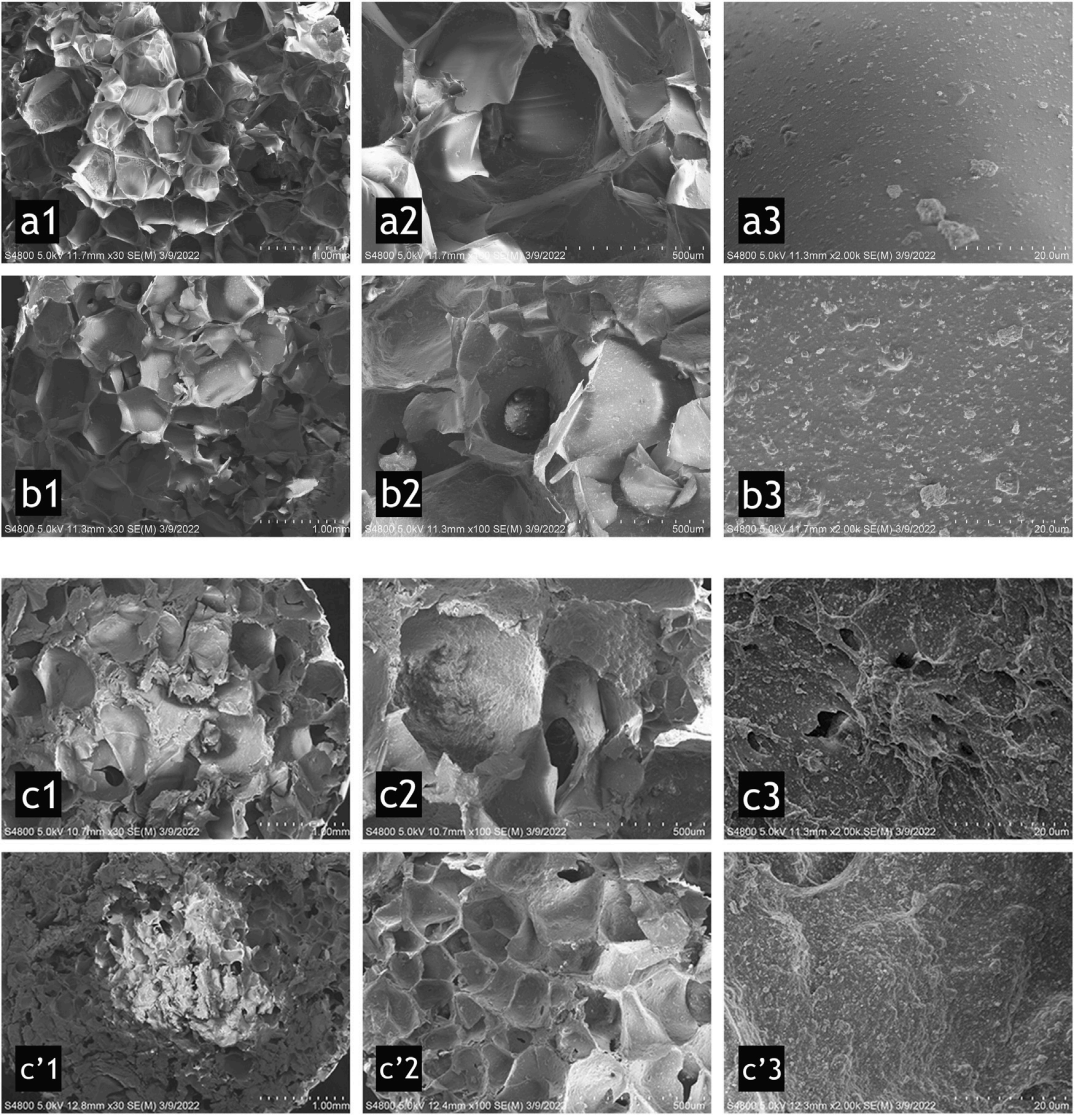
SEM micrographs for group A (a1–a3) (10% HCP), group B (b1–b3) (30% HCP), group C top (c1–c3), and bottom (c’1–c’3) (50% HCP) of the scaffold. From left to right, the scale bars represent 1.0 mm, 500 μm, and 20 μm. The porous structure was uniform in groups A and B (a1 and b1), which were different from that in group C (c1 and c’1). The pore size in c’1 was much smaller than the other groups. The pore connections were observed in b2 and c2. The inner surface of the scaffolds was gradually rougher (from a3, b3 to c3). Some small pores were observed in group C (c’3).

**FIGURE 3:**
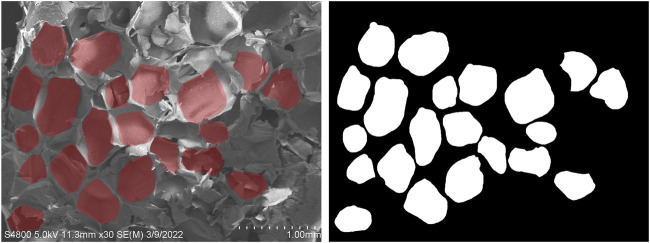
Image processing and an example of a field of vision in group B.

The micro appearance and morphology of the three groups were characterized by SEM at different magnifications, as shown in Figure 2. Groups A and B had a porous structure with uniform size. ImageJ software was used to determine the average pore size using the Feret diameter (Figure 3). Group A was 551 μm, and group B was 585 μm, which were relatively homogeneous (Figure 2 a1, b1). However, group C had a more discrete pore size (*p* < 0.05), and a nonuniform internal structure was observed, as shown in Figure 2 (c1, c’1). The pore size in the top area was approximately 553 μm, while in the bottom area it was approximately 182 μm. At ×100, connection between the pores in each group was observed (Figure 2 b2, c2). At ×2000, nano-HA and β-TCP particles were evenly distributed in the scaffolds; with the increase in the HCP content (Figure 2 a3, b3, and c3), the inner surface of the scaffolds became gradually rougher (Figure 2 c’3). The rough inner surface was conductive to protein adsorption and cell adhesion. Some small pores (approximately 3–5 μm in diameter) were observed in group C.

### 3.2 Physicochemical characterization

**FIGURE 4:**
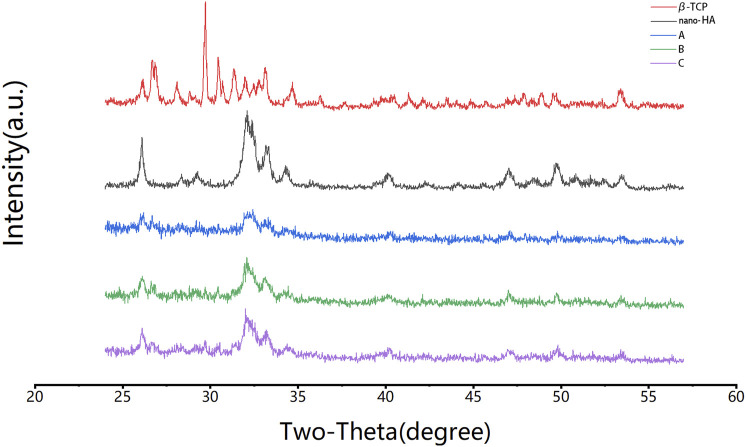
XRD analysis of the formulations. Group B and group C had diffraction peaks at 26°, 29°, 32°, and 33° 2θ, which were related to nano-HA and β-TCP, and were not observed in group A. The crystalline phases of nano-HA and β-TCP were observed.

The X-ray diffraction of group A, B, and C scaffolds is shown in Figure 4. The diffraction peak area increased with the increasing HCP content. Groups B and C showed diffraction peaks at 26°, 29°, 32°, and 33° 2θ, which were related to nano-HA and β-TCP. Group A with only 10% HCP had insufficient crystallinity. However, most of the diffraction peaks of nano-HA and β-TCP were confirmed in groups B and C, respectively. The crystalline planes of the nanocomposite showed that freeze drying with the high-speed stirring method did not change the crystalline phase of nano-HA and β-TCP.

**FIGURE 5:**
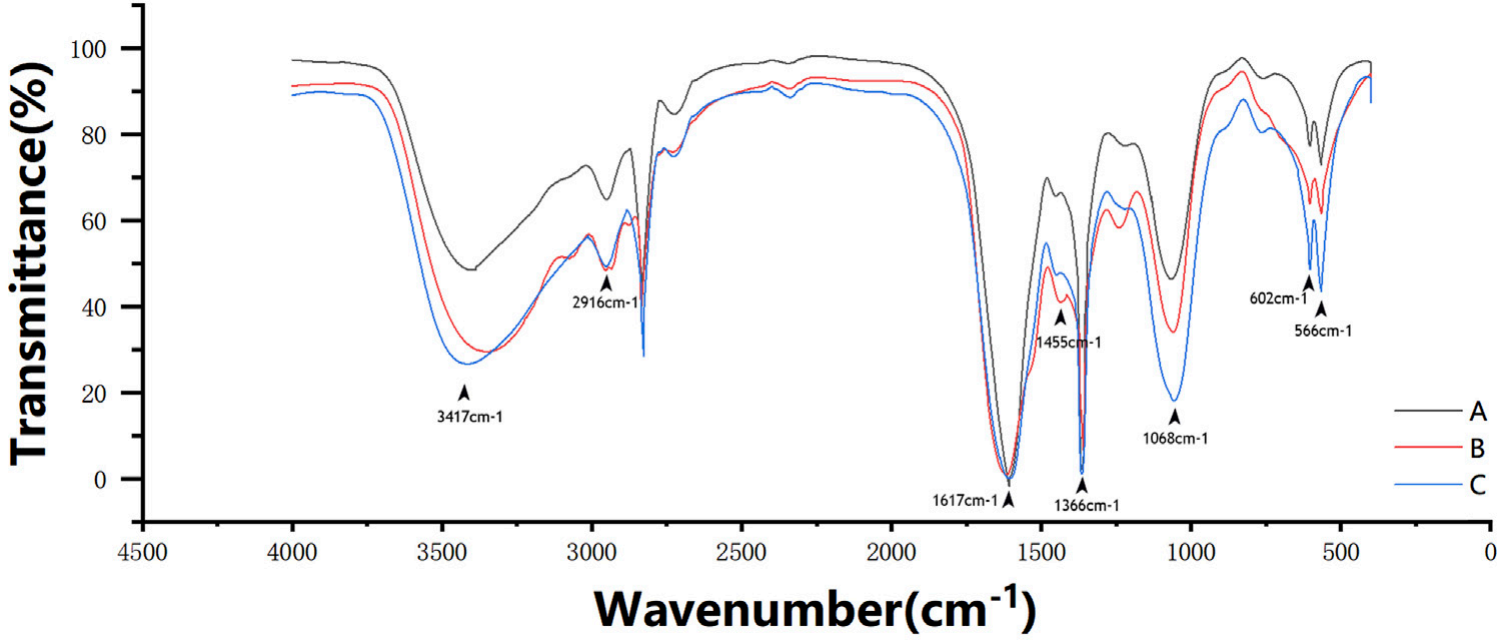
FTIR spectroscopy spectral profile of the three scaffold groups. The absorption peak at ∼3,417 cm^−1^ belongs to the –OH and –NH vibration absorptions. ∼2,916 cm^−1^ belongs to –CH, ∼1,366 cm^−1^ belongs to –CH_2_, and ∼1,068 cm^−1^ belongs to –C–O–C–. The amide I bands at ∼1,617 cm^−1^ overlaps with the amide II bands at ∼1,557 cm^−1^ to form a broad peak. The peak at ∼1,455 cm^−1^ refers to the C–N stretch. The peaks at ∼602 cm^−1^ and ∼566 cm^−1^ refer to PO_4_^3+^ from nano-HA.

The scaffolds were characterized by FTIR spectroscopy, as shown in Figure 5. The absorption peak at ∼3,417 cm^−1^ belongs to the –OH and –NH vibration absorptions in gelatin and nano-HA. The variation of this peak is related to the absorption intensity, the strength of the hydrogen bond force, or the sequence of the network structure. The peak moves from ∼3,300 cm^−1^ to the high wavenumber region with the influence of Ca in β-TCP; ∼2,916 cm^−1^ belongs to –CH, ∼1,366 cm^−1^ belongs to –CH_2_, and ∼1,068 cm^−1^ belongs to –C–O–C–, confirming the existence of CMC. The amide I bands at ∼1,617 cm^−1^ overlap with the amide II bands at ∼1,557 cm^−1^, forming a broad peak, which confirms the existence of gelatin. The peak at ∼1,455 cm^−1^ refers to the C–N stretch when glutaraldehyde crosslinked gelatin and CMC (Maji et al., 2018). The peaks at ∼602 cm^−1^ and ∼566 cm^−1^ confirm PO_4_^3+^ from nano-HA. These FTIR results indicate that the peak areas increase with the increase in the TCP content.

### 3.3 Scaffold porosity

**FIGURE 6:**
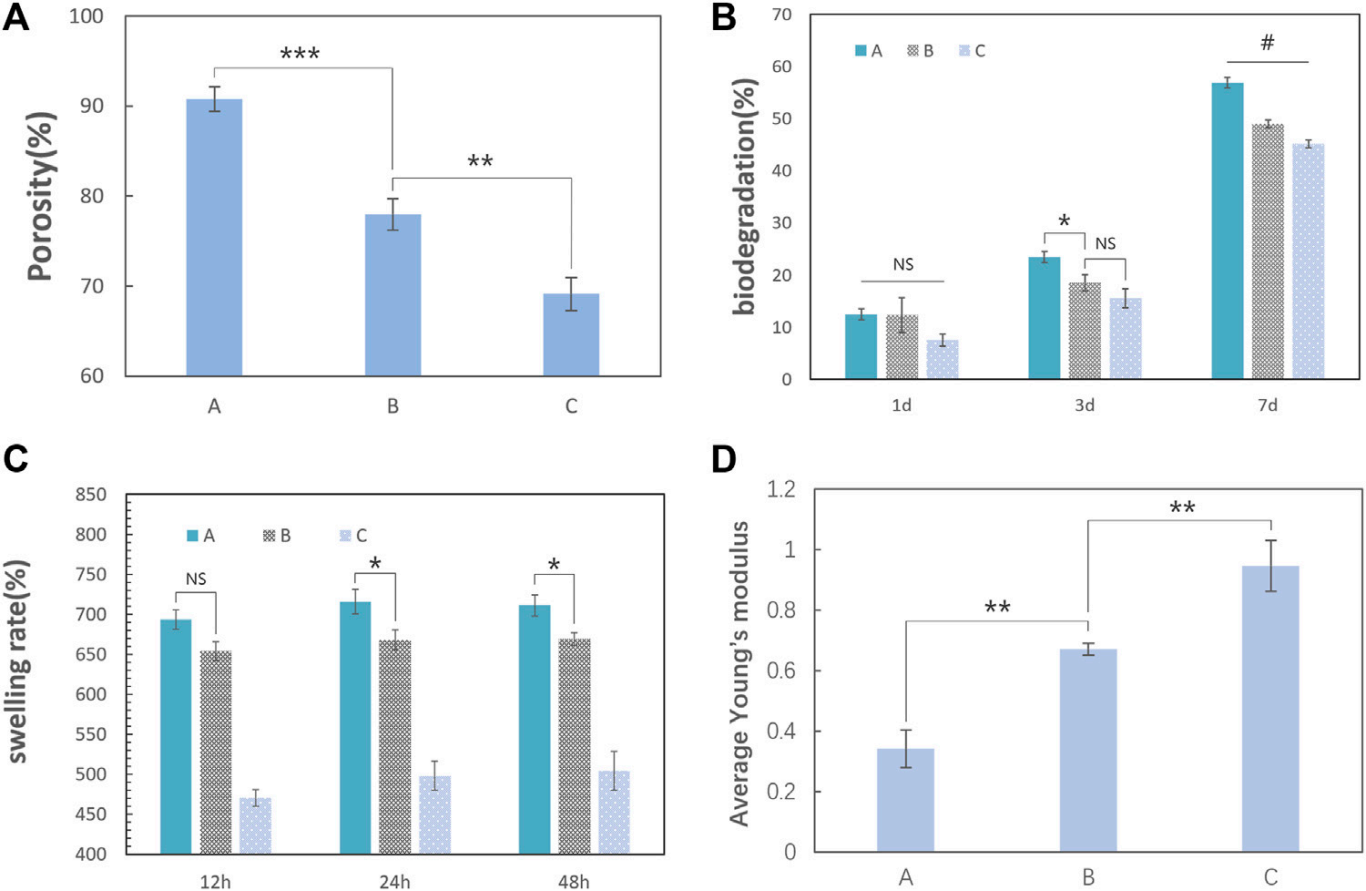
Physical properties of the nanocomposite scaffolds (the error bar represents the standard deviation of the samples). **(A)** Testing the porosity of the scaffold by the drainage method; the last column of data was the average porosity of the three groups. Group A was 91.4%, group B was 78.8%, and group C was approximately 70.0%. The porosity of the scaffold decreased gradually with the increase in the TCP content (one-way ANOVA, *F* = 130; *p* < 0.0001; *n* = 3). **(B)** Biodegradation of the three groups at 7 days. The percentage degradation values of the three groups in a type I collagenase solution for 7 days were approximately 56.9% for group A, 49.0% for group B, and 45.1% for group C. With an increase in the TCP content in the scaffold, the degradation rate on the 7th day gradually decreases (one-way ANOVA, *F* = 149.8, *p* < 0.001, and *n* = 3). **(C)** Swelling rate for the three groups in 48 h. The scaffolds had a considerable swelling ratio and reached its peak at group A (715.8%) in 24 h (one-way ANOVA, *p* < 0.05, *n* = 3). **(D)** Average Young’s modulus of the scaffold tested in dry conditions. Group A was 0.946 MPa, group B was 0.671 MPa, and group C was 0.342 MPa. Young’s modulus of the scaffolds increased with the HCP content (one-way ANOVA, *F* = 72.72, *p* < 0.0001, and *n* = 3). (“#” represents a significant difference between each group; *p < 0 .05; **p < 0.01; ***p < 0.001; ****p < 0.0001; “NS” represent no significant differences between each group.)

The porosity of the scaffold was measured using the drainage method. Although group C had an inhomogeneous internal pore structure, testing the bottom and top areas separately was difficult because it was difficult to define the line of demarcation. As a result, the average porosity was tested. Group A was 91.4%, group B was 78.8%, and group C was the least (approximately 70.0%) (Figure 6A).

### 3.4 Scaffold degradation and swelling test

Biodegradation is another important characteristic of the scaffolds for bone tissue engineering. Slow and sustained degradation of the scaffold allows new bone tissue to grow and replace the scaffold, and it will affect the quality of bone formation or lead to bone fracture if the scaffold remains for too long. Additionally, completely decomposed scaffolds avoid the need for a secondary surgery. Type I collagenase is one of the most abundant matrix metalloproteinases in mammals. The degradation rates (Figure 6B) of the three scaffolds in type I collagenase solution at 7 days were approximately 56.9% for group A, 49.0% for group B, and 45.1% for group C. Therefore, the higher the porosity, the easier will be the mass transfer for the faster degradation of the scaffold. The ability to absorb liquid substances is essential for the scaffolds in bone tissue engineering because it is a factor that determines the mass transfer of the scaffold (Maji et al., 2018). The swelling test (Figure 6C) demonstrated that the scaffolds had a considerable swelling ratio and the ratio reaching a maximum in group A (715.8%) at 24 h.

### 3.5 Mechanical test

The scaffold should maintain mechanical integrity throughout the healing process (Bharadwaz and Jayasuriya, 2020), which means that the scaffolds should maintain their original structure when squeezed by the surrounding tissue. When the scaffolds are implanted in the body and absorb tissue fluid and blood, they are mostly in a wet condition. In our research, the scaffold is soft and elastic after the *in vitro* swelling test. This suggests that our scaffold should be mainly used to repair bone defects in nonweight-bearing areas, especially in an orofacial bone defect caused by inflammation, tumors, trauma, or cysts. Because the difference in mechanical properties among the three groups in wet conditions may be difficult to compare, we used the mechanical data on the scaffolds under dry conditions to determine the scaffold that has the resistance to external forces to maintain its structure for loading cells and allowing blood vessels to grow. Young’s modulus of the scaffolds indicate that the HCP content improved the strength, with a maximum value of 0.947 MPa for group C (Figure 6D).

### 3.6 MTT assay

The results of the MTT assay in Figure 8A showed that the difference in the OD values between the control group and other three groups was not statistically significant (*p* = 0.6951), which means that the method used to crosslink gelatin and CMC had little cytotoxicity.

### 3.7 Cell proliferation assays and adhesion observation

**FIGURE 7:**
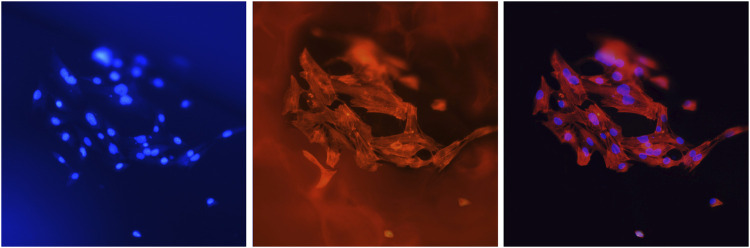
Cells (MC3T3-E1) attached to the scaffolds. Blue fluorescence shows the nuclei stained with DAPI, and red fluorescence shows the cellular F-actin stained with FITC-phalloidin. The magnification was ×400.

To ensure that most cells inoculated the scaffold material, only a small amount of complete medium was added to the 24-well plate in the initial culture stage. In this process, it was inevitable that some cells failed or died of various reasons such as insufficient nutrient supply or direct exposure to air. However, the cells remaining on the scaffold grew well (Figure 7).

**FIGURE 8:**
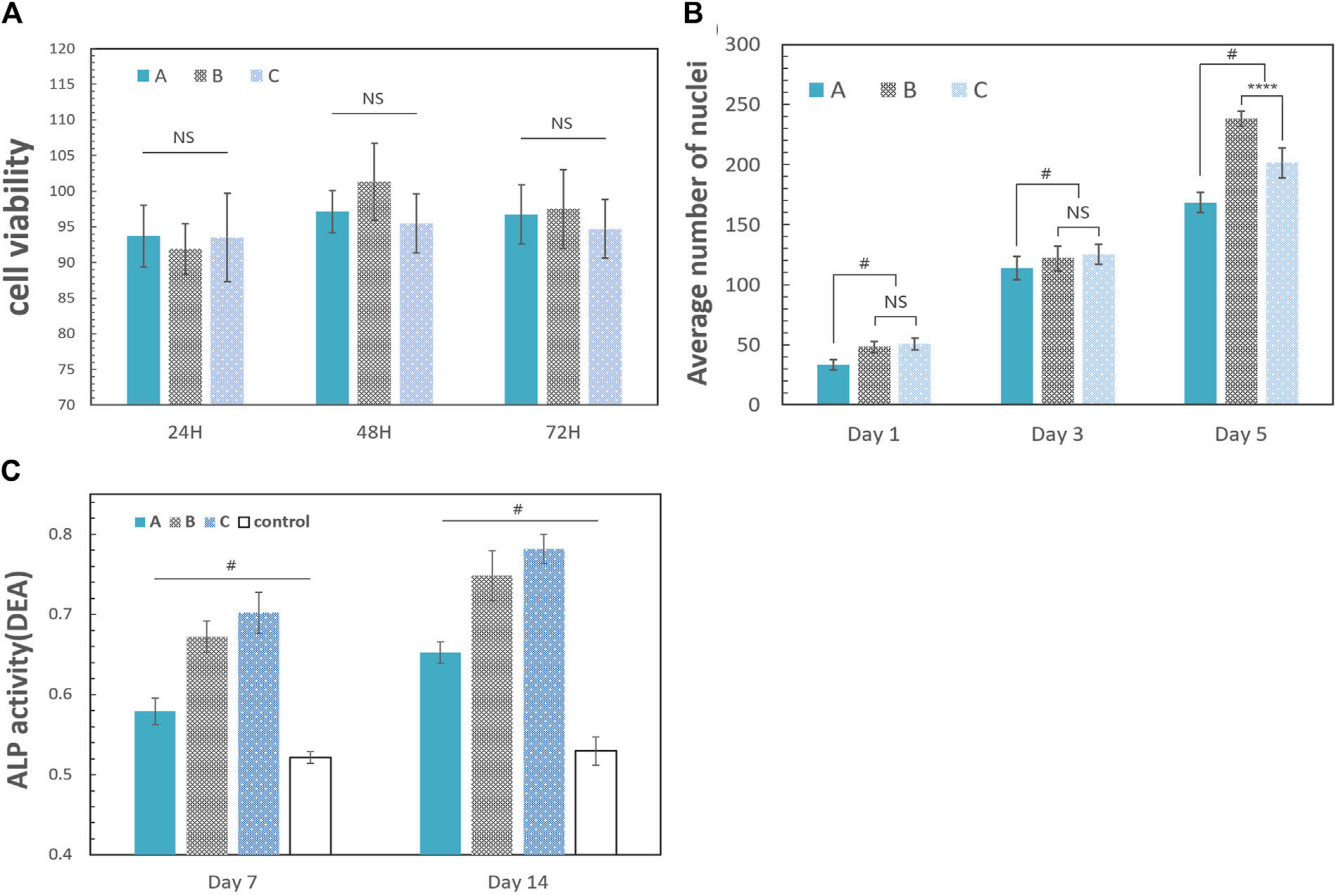
Biological properties of the scaffold. The error bar represents the standard deviation of the sample. **(A)** Results 24, 48, and 72 h after coculture with leach liquors [A: group A (10% HCP); B: group B (30% HCP); and C: group C (50% HCP)]. The difference between each group was not statistically significant (one-way ANOVA, *F* = 0.6906, *p* = 0.6951, and *n* = 5). **(B)** Cell proliferation analysis of the scaffolds. The data were the average number of nuclei after cell counting with ImageJ software. The number of proliferating cells in group A was generally fewer than the other two groups. There were no significant differences between group B and group C on the 1st and 3rd day (one-way ANOVA, for 1st day: *F* = 1.341; *p* = 0.3924 and *n* = 5; for 3rd day: *F* = 1.582; *p* = 0.5188 and *n* = 5). The cell density in group B increased rapidly from the 3rd day to the 5th day, which was more than that in group C (one-way ANOVA, *F* = 4.216; *p* < 0.0001 and *n* = 5). **(C)** ALP activity on days 7 and 14. A: group A (10% HCP); B: group B (30% HCP); C: group C (50% HCP); control: only CMC and gelatin. DEA is defined as per the manual of the ALP assay kit: the amount of ALP required to hydrolyze the chromogenic substrate (para-nitrophenyl phosphate) in diethanolamine (DEA) buffer (pH 9, 37°C), producing 1 μmol p-nitrophenol per minute, is defined as one enzyme activity unit, called one DEA (one-way ANOVA, *F* = 71.56; *p* < 0.05 and *n* = 5). (“#” represents a significant difference between each group; *p < 0.05; **p < 0.01; ***p < 0.001; ****p < 0.0001; “NS” represents no significant differences between each group).

The scaffolds in this study have a rough surface, which is conducive to protein adsorption, especially involving laminin, fibronectin, and vitronectin, and can promote the adhesion and migration of osteoblasts (Schneider et al., 2004; Gui et al., 2018; Kazimierczak et al., 2019). At the same time, a biocompatible scaffold should promote cell proliferation to ensure osteogenic differentiation. As shown in Figure 8B, the number of cells inoculated on the scaffold increased significantly. The number of proliferating cells in group A was generally fewer than that in the other two groups. There were no significant differences between group B and group C on the 1st and 3rd day, and the increasing trend of the cell density in group B and group C was faster than that in group A because of the higher HCP content. However, the decrease in cell proliferation in group C on the 5th day may be attributed to the nonuniform internal structure of the scaffold. Additionally, the reduction in pore size and low connectivity of the materials reduced the efficiency of nutrient transmission and slowed the cell growth. This effect was not influential at the early stage (the first 3 days). However, the number of cells gradually increased with time (from the 3rd day to the 5th day).

### 3.8 Alkaline phosphatase activity test

Extracellular ALP activity is an important parameter that predicts *in vivo* and *in vitro* osteogenic potential (Prins et al., 2014). Osteoblast differentiation was studied for 14 days using cell culture in leach liquors, and the results are shown in Figure 8C. On the 7th day, the ALP activity of the experimental groups was greater than the control group, and this trend continued until the 14th day. The stimulation of osteogenesis in group A was not obvious, which was related to the low HCP content in the scaffold. Although the discrepancies of groups B and C were not obvious, the ALP activity of group C was greater than that of group B on the 7th and 14th day (*p* < 0.05) because of the HCP content.

## 4 Discussion

Gelatin, a product of the partial hydrolysis of collagen, is a macromolecular hydrophilic colloid. Gelatin contains a large number of polypeptide chains in a 3D structure and has both hydrophilic and hydrophobic groups that enhance its surface activity (Liu et al., 2015; Echave et al., 2017). CMC is the carboxymethylation product of chitosan. The water solubility of CMC is significantly greater than that of chitosan because of the introduction of hydrophilic groups and carboxymethyl into the chitosan molecule, which destroys the regularity of its crystal and reduces the crystallinity (Taubner et al., 2020). Meanwhile, CMC can still retain the excellent properties of chitosan, such as biocompatibility, antibacterial behavior, promoting chondrocyte growth and hemostasis, and improving water solubility, making it widely used in regenerative medicines (Patrulea et al., 2015; Shariatinia, 2018). According to the results of FTIR spectroscopy, the peptide bonds, hydroxyls, and amino groups that play key functional roles have been retained in these scaffolds. However, the scaffolds with only natural polymers lack mechanical strength. The addition of CaPs improves mechanical properties and introduces osteoinduction, as shown by the ALP activity test, where more CaPs increase the expression of ALP in osteoblasts. Therefore, inspired by biphasic CaPs, we hope that nano-HA and β-TCP can achieve better osteointegration, osteoinduction, and osteoconduction through performance complementarity (Bouler et al., 2017). There are various methods to prepare porous osteogenic scaffold materials, such as 3D printing, electrospinning, and gas foaming technology (Kim et al., 2017). Freeze drying was selected because it is relatively mild, there is no high temperature reaction, and it will not cause thermal degradation or denaturation of the polymer in the system (Nam and Park, 1999; Abdelwahed et al., 2006). Additionally, a pore size of 200–500 μm is considered ideal for bone regeneration and vascularization (Tsuruga et al., 1997; Thavornyutikarn et al., 2014; Roseti et al., 2017). Although the freeze-drying technique can control the pore size by tuning the freezing regime, it can cause other problems such as requiring a long processing time and high energy consumption (Roseti et al., 2017). Therefore, we hoped to improve this problem by applying high-speed stirring.

The scaffolds in this study were macroporous with a high porosity and pore connectivity, as observed with SEM. The interconnected pores had a rough inner surface: at ×2,000 magnification, group C and group B had coarser inner surfaces than group A (Figure 2), which was likely related to the increase in the HCP content. The roughness of the inner surface of the hydroxyapatite scaffold will improve protein adsorption and cell adhesion (Kazimierczak et al., 2019). The Frete diameter of the scaffold pores was approximately 500 μm in groups A and B. The different scaffold components (mainly HCP) do not influence the pore size in those two groups because the scaffold’s pores were attributed to the foam caused by high-speed stirring. The porosity of the three groups of scaffolds was measured, and all had high porosity (greater than 70%). The results also showed that the porosity of the scaffold decreases with the increase in HCP, and there might have relevance between them. The degradation test results showed that the three groups of scaffolds had a good biodegradation rate with collagenase type I: with an increase in the TCP content in the scaffold, the degradation rate on the 7th day gradually decreased (*p* < 0.05). These characteristics provide available biological properties to the scaffold. However, the situation changed for group C. The pore size at the bottom of the mold was much smaller than the pores at the top, and the porosity gradually decreased with the increase in solid composition. The 5% gelatin content may not have been sufficient to envelope all of the solid components when the mechanical support was impacted with high-speed stirring. As a result, it was unable to form a uniform and stable mixture. Some small pores (approximately 3–5 μm in diameter) were also observed in group C (Figure 2 c3). Considering that the mixing time was sufficient, excess HCP was more likely to cause this phenomenon. The liquid substance (gelatin and CMC) was prevented from enveloping excessive solid matter (HCP), exposing TCP particles that fell off, and formed the micropores. As a result, when loading the raw material foam into the mold, the excessive HCP in the mixture precipitated, while the larger foam moved upward, forming a phenomenon similar to “structural delamination.” We tried to increase the concentration of gelatin to enhance the viscosity of the mixture. However, a sticky paste obstructed the foaming process and completely failed when the gelatin concentration reached 10%. Stir foaming with freeze drying may have an upper limit of the solid content, and the advantages introduced by the higher content of CaPs, such as improved mechanical properties and higher osteogenic differentiation activity, are still a goal of bone tissue engineering (Bose et al., 2012; Roseti et al., 2017; Kazimierczak et al., 2019). In addition, although the scaffolds manufactured in this study have high mechanical strength when dried *in vitro*, they were soft and elastic under wet conditions *in vivo*, which may not be suitable for bone defect areas with high stress such as a segmental defect of the long bone or the mandibular bone (Dumic-Cule et al., 2015; Henkel et al., 2021). Therefore, how to improve the mechanical strength of the scaffold manufactured with this method still needs further research and exploration.

## 5 Conclusion

The new degradable bone scaffold of gelatin/CMC loaded with HCP formed by freeze drying and stir foaming provides an ideal composite for bone tissue engineering. The scaffolds promote osteogenic differentiation, with group B (30% HCP) demonstrating the most promising characteristics. We hypothesize that the scaffolds may have great potential in bone regeneration.

## Data Availability

The raw data supporting the conclusion of this article will be made available by the authors, without undue reservation.

## References

[B1] Abdelwahed W. Degobert G. Stainmesse S. Fessi H. (2006). Freeze-drying of nanoparticles: Formulation, process and storage considerations. Adv. Drug Deliv. Rev. 58, 1688–1713. 10.1016/j.addr.2006.09.017 17118485

[B2] Abdul Halim N. A. Hussein M. Z. Kandar M. K. (2021). Nanomaterials-upconverted hydroxyapatite for bone tissue engineering and a platform for drug delivery. Int. J. Nanomedicine 16, 6477–6496. 10.2147/ijn.s298936 34584412 PMC8464594

[B3] Agarwal T. Narayan R. Maji S. Behera S. Kulanthaivel S. Maiti T. K. (2016). Gelatin/Carboxymethyl chitosan based scaffolds for dermal tissue engineering applications. Int. J. Biol. Macromol. 93, 1499–1506. 10.1016/j.ijbiomac.2016.04.028 27086289

[B4] Bharadwaz A. Jayasuriya A. C. (2020). Recent trends in the application of widely used natural and synthetic polymer nanocomposites in bone tissue regeneration. Mater. Sci. Eng. C 110, 110698. 10.1016/j.msec.2020.110698 PMC7433904 32204012

[B5] Bohner M. Santoni B. L. G. DöBELIN N. (2020). β-tricalcium phosphate for bone substitution: Synthesis and properties. Acta Biomater. 113, 23–41. 10.1016/j.actbio.2020.06.022 32565369

[B6] Bose S. Roy M. Bandyopadhyay A. (2012). Recent advances in bone tissue engineering scaffolds. Trends Biotechnol. 30, 546–554. 10.1016/j.tibtech.2012.07.005 22939815 PMC3448860

[B7] Bouler J. M. Pilet P. Gauthier O. Verron E. (2017). Biphasic calcium phosphate ceramics for bone reconstruction: A review of biological response. Acta Biomater. 53, 1–12. 10.1016/j.actbio.2017.01.076 28159720

[B8] Buck D. W. Dumanian G. A. (2012). Bone biology and physiology: Part I. The fundamentals. Plastic Reconstr. Surg. 129, 1314–1320. 10.1097/prs.0b013e31824eca94 22634648

[B9] Catalan K. N. Corrales T. P. Forero J. C. Romero C. P. Acevedo C. A. (2019). Glass transition in crosslinked nanocomposite scaffolds of gelatin/chitosan/hydroxyapatite. . Polymers (Basel), 11(4):642. 10.3390/polym11040642 PMC6523647 30970604

[B10] Chen P. Liu L. Pan J. Mei J. Li C. Zheng Y. (2019). Biomimetic composite scaffold of hydroxyapatite/gelatin-chitosan core-shell nanofibers for bone tissue engineering. Mater. Sci. Eng. C 97, 325–335. 10.1016/j.msec.2018.12.027 30678918

[B11] Cho J. S. Ko Y. N. Koo H. Y. Kang Y. C. (2010). Synthesis of nano-sized biphasic calcium phosphate ceramics with spherical shape by flame spray pyrolysis. J. Mat. Sci. Mat. Med. 21, 1143–1149. 10.1007/s10856-009-3980-1 20052521

[B12] Da Silva Brum I. De Carvalho J. J. Da Silva Pires J. L. De Carvalho M. A. A. Dos Santos L. B. F. Elias C. N. (2019). Nanosized hydroxyapatite and β-tricalcium phosphate composite: Physico-chemical, cytotoxicity, morphological properties and *in vivo* trial. Sci. Rep. 9, 19602. 10.1038/s41598-019-56124-4 31863078 PMC6925105

[B13] Da Silva Brum I. Frigo L. Goncalo Pinto Dos Santos P. Nelson Elias C. Da Fonseca G. Jose De Carvalho J. (2021). Performance of nano-hydroxyapatite/beta-tricalcium phosphate and xenogenic hydroxyapatite on bone regeneration in rat calvarial defects: Histomorphometric, immunohistochemical and ultrastructural analysis. Int. J. Nanomedicine 16, 3473–3485. 10.2147/ijn.s301470 34040373 PMC8140889

[B14] Dumic-Cule I. Pecina M. Jelic M. Jankolija M. Popek I. Grgurevic L. (2015). Biological aspects of segmental bone defects management. Int. Orthop. 39, 1005–1011. 10.1007/s00264-015-2728-4 25772279

[B15] Ebrahimi M. Botelho M. G. Dorozhkin S. V. (2017). Biphasic calcium phosphates bioceramics (HA/TCP): Concept, physicochemical properties and the impact of standardization of study protocols in biomaterials research. Mater. Sci. Eng. C 71, 1293–1312. 10.1016/j.msec.2016.11.039 27987685

[B16] Echave M. C. Saenz Del Burgo L. Pedraz J. L. Orive G. (2017). Gelatin as biomaterial for tissue engineering. Curr. Pharm. Des. 23, 3567–3584. 10.2174/0929867324666170511123101 28494717

[B17] Filippi M. Born G. Chaaban M. Scherberich A. (2020). Natural polymeric scaffolds in bone regeneration. Front. Bioeng. Biotechnol. 8, 474. 10.3389/fbioe.2020.00474 32509754 PMC7253672

[B18] Ghiasi B. Sefidbakht Y. Mozaffari-Jovin S. Gharehcheloo B. Mehrarya M. Khodadadi A. (2020). Hydroxyapatite as a biomaterial - a gift that keeps on giving. Drug Dev. Ind. Pharm. 46, 1035–1062. 10.1080/03639045.2020.1776321 32476496

[B19] Gui N. Xu W. Myers D. E. Shukla R. Tang H. P. Qian M. (2018). The effect of ordered and partially ordered surface topography on bone cell responses: A review. Biomater. Sci. 6, 250–264. 10.1039/c7bm01016h 29313536

[B20] Han F. Dong Y. Su Z. Yin R. Song A. Li S. (2014). Preparation, characteristics and assessment of a novel gelatin-chitosan sponge scaffold as skin tissue engineering material. Int. J. Pharm. X. 476, 124–133. 10.1016/j.ijpharm.2014.09.036 25275938

[B21] Henkel J. Medeiros Savi F. Berner A. Fountain S. Saifzadeh S. Steck R. (2021). Scaffold-guided bone regeneration in large volume tibial segmental defects. Bone 153, 116163. 10.1016/j.bone.2021.116163 34461285

[B22] Jensen S. S. Bornstein M. M. Dard M. Bosshardt D. D. Buser D. (2009). Comparative study of biphasic calcium phosphates with different HA/TCP ratios in mandibular bone defects. A long-term histomorphometric study in minipigs. J. Biomed. Mat. Res. 90, 171–181. 10.1002/jbm.b.31271 19085941

[B23] Kazimierczak P. Benko A. Nocun M. Przekora A. (2019). Novel chitosan/agarose/hydroxyapatite nanocomposite scaffold for bone tissue engineering applications: Comprehensive evaluation of biocompatibility and osteoinductivity with the use of osteoblasts and mesenchymal stem cells. Int. J. Nanomedicine 14, 6615–6630. 10.2147/ijn.s217245 31695360 PMC6707379

[B24] Kim H. D. Amirthalingam S. Kim S. L. Lee S. S. Rangasamy J. Hwang N. S. (2017). Bone tissue engineering: Biomimetic materials and fabrication approaches for bone tissue engineering (adv. Healthcare mater. 23/2017). Adv. Healthc. Mat. 6, 1770120. 10.1002/adhm.201770120 29171714

[B25] Liu D. Nikoo M. Boran G. Zhou P. Regenstein J. M. (2015). Collagen and gelatin. Annu. Rev. Food Sci. Technol. 6, 527–557. 10.1146/annurev-food-031414-111800 25884286

[B26] Lowe B. Hardy J. G. Walsh L. J. (2020). Optimizing nanohydroxyapatite nanocomposites for bone tissue engineering. ACS Omega 5, 1–9. 10.1021/acsomega.9b02917 31956745 PMC6963893

[B27] Maji S. Agarwal T. Das J. Maiti T. K. (2018). Development of gelatin/carboxymethyl chitosan/nano-hydroxyapatite composite 3D macroporous scaffold for bone tissue engineering applications. Carbohydr. Polym. 189, 115–125. 10.1016/j.carbpol.2018.01.104 29580388

[B28] Montaser A. S. Jlassi K. Ramadan M. A. Sleem A. A. Attia M. F. (2021). Alginate, gelatin, and carboxymethyl cellulose coated nonwoven fabrics containing antimicrobial AgNPs for skin wound healing in rats. Int. J. Biol. Macromol. 173, 203–210. 10.1016/j.ijbiomac.2021.01.123 33484799

[B29] Nabavinia M. Khoshfetrat A. B. Naderi-Meshkin H. (2019). Nano-hydroxyapatite-alginate-gelatin microcapsule as a potential osteogenic building block for modular bone tissue engineering. Mater. Sci. Eng. C 97, 67–77. 10.1016/j.msec.2018.12.033 30678955

[B30] Nam Y. S. Park T. G. (1999). Biodegradable polymeric microcellular foams by modified thermally induced phase separation method. Biomaterials 20, 1783–1790. 10.1016/s0142-9612(99)00073-3 10509188

[B31] Noor Z. (2013). Nanohydroxyapatite application to osteoporosis management. J. Osteoporos. 2013, 1–6. 10.1155/2013/679025 PMC3830883 24288653

[B32] Oryan A. Alidadi S. Bigham-Sadegh A. Meimandi-Parizi A. (2017). Chitosan/gelatin/platelet gel enriched by a combination of hydroxyapatite and beta-tricalcium phosphate in healing of a radial bone defect model in rat. Int. J. Biol. Macromol. 101, 630–637. 10.1016/j.ijbiomac.2017.03.148 28363647

[B33] Patrulea V. Ostafe V. Borchard G. Jordan O. (2015). Chitosan as a starting material for wound healing applications. Eur. J. Pharm. Biopharm. 97, 417–426. 10.1016/j.ejpb.2015.08.004 26614560

[B34] Prins H. J. Braat A. K. Gawlitta D. Dhert W. J. Egan D. A. Tijssen-Slump E. (2014). *In vitro* induction of alkaline phosphatase levels predicts *in vivo* bone forming capacity of human bone marrow stromal cells. Stem Cell Res. 12, 428–440. 10.1016/j.scr.2013.12.001 24384458

[B35] Roseti L. Parisi V. Petretta M. Cavallo C. Desando G. Bartolotti I. (2017). Scaffolds for bone tissue engineering: State of the art and new perspectives. Mater. Sci. Eng. C 78, 1246–1262. 10.1016/j.msec.2017.05.017 28575964

[B36] Samirah A. U. Budiatin A. S. Mahyudin F. Khotib J. (2021). Fabrication and characterization of bovine hydroxyapatite-gelatin-alendronate scaffold cross-linked by glutaraldehyde for bone regeneration. J. Basic Clin. Physiol. Pharmacol. 32, 555–560. 10.1515/jbcpp-2020-0422 34214349

[B37] Schneider G. B. English A. Abraham M. Zaharias R. Stanford C. Keller J. (2004). The effect of hydrogel charge density on cell attachment. Biomaterials 25, 3023–3028. 10.1016/j.biomaterials.2003.09.084 14967535

[B38] Shao H. Liu A. Ke X. Sun M. He Y. Yang X. (2017). 3D robocasting magnesium-doped wollastonite/TCP bioceramic scaffolds with improved bone regeneration capacity in critical sized calvarial defects. J. Mat. Chem. B 5, 2941–2951. 10.1039/c7tb00217c 32263987

[B39] Shariatinia Z. (2018). Carboxymethyl chitosan: Properties and biomedical applications. Int. J. Biol. Macromol. 120, 1406–1419. 10.1016/j.ijbiomac.2018.09.131 30267813

[B40] Taubner T. Marounek M. Synytsya A. (2020). Preparation and characterization of hydrophobic and hydrophilic amidated derivatives of carboxymethyl chitosan and carboxymethyl β-glucan. Int. J. Biol. Macromol. 163, 1433–1443. 10.1016/j.ijbiomac.2020.07.257 32738322

[B41] Thavornyutikarn B. Chantarapanich N. Sitthiseripratip K. Thouas G. A. Chen Q. (2014). Bone tissue engineering scaffolding: Computer-aided scaffolding techniques. Prog. Biomater. 3, 61–102. 10.1007/s40204-014-0026-7 26798575 PMC4709372

[B42] Tsuruga E. Takita H. Itoh H. Wakisaka Y. Kuboki Y. (1997). Pore size of porous hydroxyapatite as the cell-substratum controls BMP-induced osteogenesis. J. Biochem. 121, 317–324. 10.1093/oxfordjournals.jbchem.a021589 9089406

[B43] Venkatesan J. Kim S. K. (2014). Nano-hydroxyapatite composite biomaterials for bone tissue engineering-a review. J. Biomed. Nanotechnol. 10, 3124–3140. 10.1166/jbn.2014.1893 25992432

[B44] Vieira S. Vial S. Reis R. L. Oliveira J. M. (2017). Nanoparticles for bone tissue engineering. Biotechnol. Prog. 33, 590–611. 10.1002/btpr.2469 28371447

[B45] Wu T. Yu S. Chen D. Wang Y. (2017). Bionic design, materials and performance of bone tissue scaffolds. Mater. (Basel) 10, 1187. 10.3390/ma10101187 PMC5666993 29039749

[B46] Xu Y. Zhang Z. Wang H. Zhong W. Sun C. Sun W. (2021). Zoledronic acid-loaded hybrid hyaluronic acid/polyethylene glycol/nano-hydroxyapatite nanoparticle: Novel fabrication and safety verification. Front. Bioeng. Biotechnol. 9, 629928. 10.3389/fbioe.2021.629928 33659241 PMC7917242

[B47] Yamada S. Heymann D. Bouler J. M. Daculsi G. (1997). Osteoclastic resorption of biphasic calcium phosphate ceramic *in vitro* . J. Biomed. Mat. Res. 37, 346–352. 10.1002/(sici)1097-4636(19971205)37:3<346::aid-jbm5>3.0.co;2-l 9368139

[B48] Yan Y. Chen H. Zhang H. Guo C. Yang K. Chen K. (2019). Vascularized 3D printed scaffolds for promoting bone regeneration. Biomaterials 190-191, 97–110. 10.1016/j.biomaterials.2018.10.033 30415019

