# Supplementary material for: A novel gelatin/carboxymethyl chitosan/nano-hydroxyapatite/β-tricalcium phosphate biomimetic nanocomposite scaffold for bone tissue engineering applications
Source: Front Chem. 2022 Sep 8;10:958420. doi: 10.3389/fchem.2022.958420 (PMC9493496; doi:10.3389/fchem.2022.958420)
Supplement: Supplementary file 1 [file Table1.DOCX]

Table.1

Table.2

Table.3

Table.4

Table.5

Table.6

Table.7
